# Microbiome of Odontogenic Abscesses

**DOI:** 10.3390/microorganisms9061307

**Published:** 2021-06-16

**Authors:** Sebastian Böttger, Silke Zechel-Gran, Daniel Schmermund, Philipp Streckbein, Jan-Falco Wilbrand, Michael Knitschke, Jörn Pons-Kühnemann, Torsten Hain, Markus Weigel, Hans-Peter Howaldt, Eugen Domann, Sameh Attia

**Affiliations:** 1 Department of Oral and Maxillofacial Surgery, Justus-Liebig-University Giessen, University Hospital Giessen and Marburg, Giessen, D-35392 Giessen, Germany; Daniel.Schmermund@uniklinikum-giessen.de (D.S.); Philipp.Streckbein@uniklinikum-giessen.de (P.S.); Jan-Falco.Wilbrand@uniklinikum-giessen.de (J.-F.W.); Michael.Knitschke@uniklinikum-giessen.de (M.K.); HP.Howaldt@uniklinikum-giessen.de (H.-P.H.); sameh.attia@dentist.med.uni-giessen.de (S.A.); 2 Institute of Medical Microbiology, Justus-Liebig-University Giessen, D-35392 Giessen, Germany; Silke.Zechel@mikrobio.med.uni-giessen.de (S.Z.-G.); Torsten.Hain@mikrobio.med.uni-giessen.de (T.H.); Markus.Weigel@mikrobio.med.uni-giessen.de (M.W.); 3 Institute of Medical Informatics, Justus-Liebig-University Giessen, D-35392 Giessen, Germany; Joern.Pons@informatik.med.uni-giessen.de; 4 German Center for Infection Research (DZIF), Justus-Liebig-University Giessen, Partner Site Giessen-Marburg-Langen, D-35392 Giessen, Germany; Eugen.Domann@mikrobio.med.uni-giessen.de; 5 Institute of Hygiene and Environmental Medicine, Justus-Liebig-University Giessen, D-35392 Giessen, Germany

**Keywords:** oral microbiome, odontogenic abscess, 16S rRNA gene analysis, polymicrobial infection, anaerobic infection

## Abstract

Severe odontogenic abscesses are regularly caused by bacteria of the physiological oral microbiome. However, the culture of these bacteria is often prone to errors and sometimes does not result in any bacterial growth. Furthermore, various authors found completely different bacterial spectra in odontogenic abscesses. Experimental 16S rRNA gene next-generation sequencing analysis was used to identify the microbiome of the saliva and the pus in patients with a severe odontogenic infection. The microbiome of the saliva and the pus was determined for 50 patients with a severe odontogenic abscess. Perimandibular and submandibular abscesses were the most commonly observed diseases at 15 (30%) patients each. Polymicrobial infections were observed in 48 (96%) cases, while the picture of a mono-infection only occurred twice (4%). On average, 31.44 (±12.09) bacterial genera were detected in the pus and 41.32 (±9.00) in the saliva. In most cases, a predominantly anaerobic bacterial spectrum was found in the pus, while saliva showed a similar oral microbiome to healthy individuals. In the majority of cases, odontogenic infections are polymicrobial. Our results indicate that these are mainly caused by anaerobic bacterial strains and that aerobic and facultative anaerobe bacteria seem to play a more minor role than previously described by other authors. The 16S rRNA gene analysis detects significantly more bacteria than conventional methods and molecular methods should therefore become a part of routine diagnostics in medical microbiology.

## 1. Introduction

Odontogenic abscesses are among the most common inflammatory diseases in the field of oral and maxillofacial surgery. Most of these abscesses are localized and can be successfully treated simply by abscess incision intraorally. Larger abscesses often show a tendency to spread and are usually incised extraorally using general anesthesia [1]. If left untreated, such abscesses can cause severe local and systemic complications and can even lead to death [1,2,3].

Although many extensive abscesses can be successfully treated by incision alone [4,5], adjuvant antibiotic therapy is often used [6], especially if the abscessing inflammation is medial to the mandibular bone [7]. Such an antibiotic therapy is usually carried out as a calculated therapy, as the cultural assessment of the pathogens usually takes a few days and an antibiogram (antibiotic susceptibilities and resistances) is only available after some days. However, in those patients with exceptionally severe progression of the infection, a correct antibiogram can be decisive for the further course of the disease and ultimately also for the patient’s outcome [8]. The determination of the causative bacteria is therefore an important component in the diagnosis of odontogenic abscesses, especially if there are indications of an extension or a complicated course of the disease at the beginning of the therapy [9].

However, microbiological diagnostics have weaknesses. It is not always possible to sufficiently identify the causative pathogens by culture-based methods alone [10]—some authors even speak of culture-negative odontogenic abscesses if it is not possible to cultivate any pathogens at all [11]. Recent detection methods, not based on culturing, and particularly 16S rRNA gene analysis with subsequent gene sequencing, have shown that many bacteria of the oral cavity can only be determined with difficulty or even not at all by culture-based methods [3,12,13]. It has also not yet been possible to determine which bacteria of the oral and pharyngeal microbiome are exactly responsible for the acute inflammatory process and if the oral microbiome in these patients is composed differently compared to healthy people.

With advancements in sequencing technology, and particularly in next-generation sequencing-based technologies, the identification of microorganisms and community profiling has become increasingly simple and increasingly effective [14]. With these methods, massive deoxyribonucleic acid (DNA) sequencing has become possible with a much higher throughput compared to older methods, so that a large number of samples can be effectively studied at the same time [15]. Numerous authors have pointed out that previously indistinguishable hidden bacterial species can also be fully detected in this way [10,16].

With regard to extensive odontogenic abscesses, which require inpatient therapy and can occasionally assume life-threatening dimensions, the following questions therefore arise:

1. Does the oral microbiome of these patients show differences compared to the oral microbiome of healthy patients described in the literature?
2. Which bacteria can be detected in pus using 16S rRNA gene next-generation sequencing analysis?

These questions will be addressed in the present study based on patients who were treated for extensive odontogenic abscesses in a University Hospital Department for Oral and Maxillofacial Surgery.

## 2. Materials and Methods

Fifty patients treated for severe odontogenic abscesses between October 2016 and March 2017 in the Department of Oral and Maxillofacial Surgery of the university hospital Giessen and Marburg, Location Giessen, Germany, were included in the study. For the purpose of the study, a saliva sample was taken from the patients prior to the abscess incision in order to determine the oral microbiome. During abscess incision, pus was obtained from the incised abscess. Both samples, saliva and pus, were initially frozen at −80 °C for later evaluation. The patients gave their written consent before the study and the study was authorized by the local Ethics Committee of the Medical Faculty of the Justus-Liebig-University Giessen (Vote 191/16).

The determination of the microbiome was carried out around three months after the collection of the last sample with 16S rRNA amplicon sequencing and bioinformatics analysis [15,17]. First, bacterial DNA was extracted from the initially frozen pus and saliva. Then, the variable area “V4” of the 16S rRNA genes was amplified by polymerase chain reaction (PCR) using primers in the conserved flanking areas with adapters. The resulting amplicons of an approximate length of 350 to 370 base pairs (bps) were processed for next-generation sequencing using the Illumina MiSeq System (San Diego, CA, USA). After sequencing, bioinformatics analysis was performed.

### 2.1. Nucleic Acid Extraction of Samples, Library Construction, 16S rRNA Amplicon Sequencing

For nucleic acid extraction, 1 mL saliva, 0.5 mL pus and negative controls of water and reaction mixtures were used. To determine the quality of the experiments, a mock community was applied [18].

Samples were centrifuged in 1.5 mL Eppendorf tubes (13,000 rpm, 10 min). Pellets were resuspended in 150 µL lysis buffer (25 mM Tris, pH 8.0; 25 mM EDTA, pH 8.0; 1% Triton ×100). The solution was treated with 20 µL Proteinase K (20 mg/mL, Ambion, Oberursel, Germany) at 55 °C for 2 h and the enzyme was subsequently inactivated at 90 °C for 30 min. After further incubation with 50 µL lysozyme (20 mg/mL, Merck, Darmstadt, Germany), 10 µL lysostaphin (5 U/µL, Sigma, St. Louis, MO, USA) and 10 µL mutanolysin (5 U/µL, Sigma, St. Louis, MO, USA) at 37 °C for 30 min, DNA was extracted by using glass beads and the Power Lyzer DNA Isolation Kit from MoBio as recommended by the vendor (MoBio Laboratories, Carlsbad, CA, USA). DNA of all samples was eluted in 100 µL of nuclease-free water and concentration was determined using Qubit Fluorometric Quantitation (Thermo Fisher Scientific, Waltham, MA, USA). The V4 region of 16S rRNA gene was amplified using adapter forward primer 5′-TCGTCGGCAGCGTCAGATGTGTATAAGAGACAGGTGCCAGCMGCCGCGGTAA-3′, and adapter reverse primer 5′-GTCTCGTGGGCTCGGAGATGTGTATAAGAGACAGGGTACHVGGGTWTCTAAT-3′and the 2× Kapa HiFi HotStart Ready Mix (Kapa Biosystems, Wilmington, MA, USA). Amplification profile comprised an initial heating step at 95 °C for 3 min, 25 cycles of denaturation at 95 °C for 30 s, annealing at 55 °C for 30 s, elongation at 72 °C for 30 s and a final elongation step at 72 °C for 5 min. PCR products were purified with Agencourt AMPure XP system as recommended by the vendor (Beckman Coulter, Brea, CA, USA). Size, purity and concentration of amplicons were determined using the Agilent Bioanalyzer as recommended by the vendor (Agilent Technologies, Santa Clara, CA, USA). The index PCR was performed using the Nextera index Kit v2 Set B as recommended by the vendor (Illumina, San Diego, CA, USA). The quality of the index PCR was determined as described above for the adapter PCR. The library was adjusted to 3 pM, and the flow cell was prepared and loaded according to the Reagent Preparation Guide of MiSeq Reagent Kit v2 and sequenced as recommended by the vendor (Illumina).

### 2.2. Bioinformatics Analysis

Microbiome analysis was performed as previously described [19]. In brief, paired end sequence reads were joined and primer sequences were removed. Saliva sequence reads varied between 8465 and 309,076 and pus reads varied between 28,698 and 751,847 per sample. Reads with ambiguous base calls or with homopolymers longer than eight nucleotides were removed and duplicates were merged and aligned against the SILVA-bases bacterial reference alignment [20]. Applying Mothur implementation of the uchime algorithm, chimeric reads were removed, taxonomy was assigned and non-bacterial reads were removed from the analysis. Operational taxonomic units (OTU) were generated and taxonomy was reassigned using Mothur. In preparation for the analysis, an OTU table in biom format was created.

### 2.3. Statistical Analysis

Statistical analysis was carried out with Microsoft Excel (Redmond, WA, USA) and the statistical software R-4.0.4 (R Core Team, 2021, Vienna, Austria). For calculation of frequencies, we considered a phylotype to be abundant if it contributed to at least 0.01% of the microbiome. Lilliefors (Kolmogorov–Smirnov) test (R package: nortest) was applied to test for normality distribution. In addition, histograms were used to visualize alpha-diversity in the saliva and pus samples. To describe the composition of the microbiomes, means, standard deviations and medians of the relative frequencies of the reads were detected and shown in a table and in pie charts. Thus, a median microbiome was calculated for saliva and pus samples to show the median composition of the saliva and pus microbiomes. Alpha diversity was further illustrated with heatmaps directly contrasting the relative frequencies of reads in saliva and pus. Hierarchical clustering (method: ward with Euclidean distance) and principal component analysis (PCA) were used to describe beta diversity among the saliva and pus samples. Heatmaps, hierarchical clustering and PCA were based on the relative frequency of the reads of the most frequent bacterial genera (sum of rows > 20%). This means that the sum of the relative abundances in a row of the heatmaps must be more than 20% for the corresponding bacterial genus to be displayed.

Heatmaps and hierarchical clustering were performed by the R package pheatmap, and PCA was visualized by biplots using the R package ggfortify.

## 3. Results

**Figure 1:**
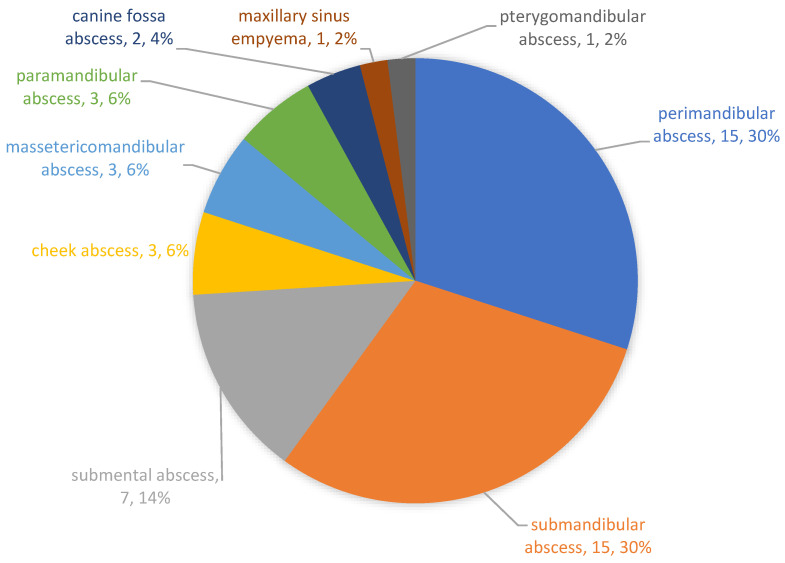
Frequency of the observed abscesses. The most common abscesses were the perimandibular and the submandibular abscess.

In the observation period from October 2016 to March 2017, a total of 50 patients with a severe odontogenic abscess were treated with incision and drainage at the University Hospital. In addition to the normal microbiological standard examination (microbiological smear and culture), a sample of saliva and drained pus was obtained from all 50 patients. Among them, 34 patients (68%) were male and 16 patients (32%) were female. The mean age was 47.42 years (standard deviation: 19.59 years). The Lilliefors test showed that the distribution of the patients’ age did not differ significantly from that which was normally distributed (*p* = 0.415). The most frequently observed abscesses were perimandibular and submandibular, occurring in 15 patients (30%) each. Figure 1 shows the frequency of the observed abscesses.

**Figure 2:**
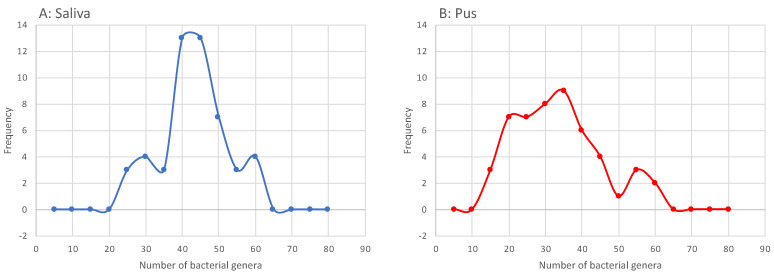
Histogram of the counted bacterial genera in the saliva (**A**) and the pus (**B**) to describe alpha diversity. The occurrence of a genus was counted if the genus contributed to at least 0.01% of the microbiome.

Furthermore, 16S rRNA gene analysis revealed the microbiome of the saliva and the drained pus. As in other studies, an abundance of bacterial genera was found, both in saliva and pus. We considered a phylotype to be abundant if it contributed to at least 0.01% of the microbiome. Using this threshold, a mean of 41.32 (±9.00) bacterial genera was found in the saliva and a mean of 31.44 (±12.09) was found in the pus. Figure 2 shows the distribution of the counted bacterial genera in the saliva and the pus. The Lilliefors test showed that the distribution of the number of bacterial genera did not differ significantly from that which was normally distributed (saliva: *p* = 0.303 and pus: *p* = 0.321). The two-tailed paired-samples *t*-test for means showed that the number of bacterial genera was significantly higher in the saliva than in the pus (*p* < 0.0001).

**Figure 3:**
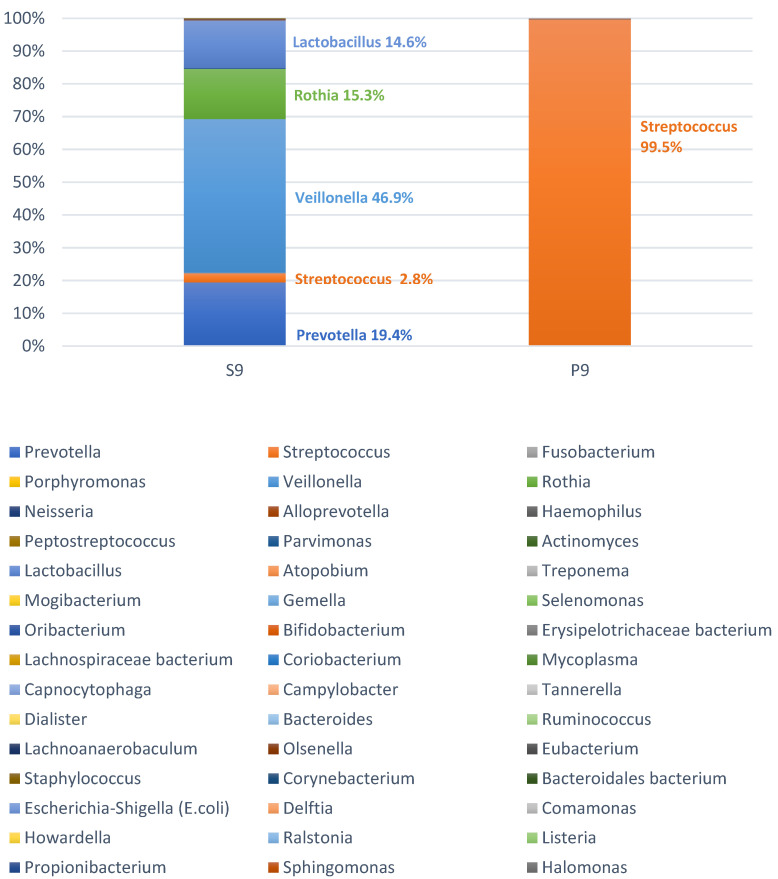
Microbiome of the saliva (S9) and the pus (P9) of patient no. 9. Herein, 99.5% of the reads belong to the genus *Streptococcus* and thus show the picture of a mono-infection. The legend contains all detected bacterial genera of the two related samples. The order in the legend corresponds to the total abundance in saliva and pus of all 50 samples (decreasing from left to right).

**Figure 4:**
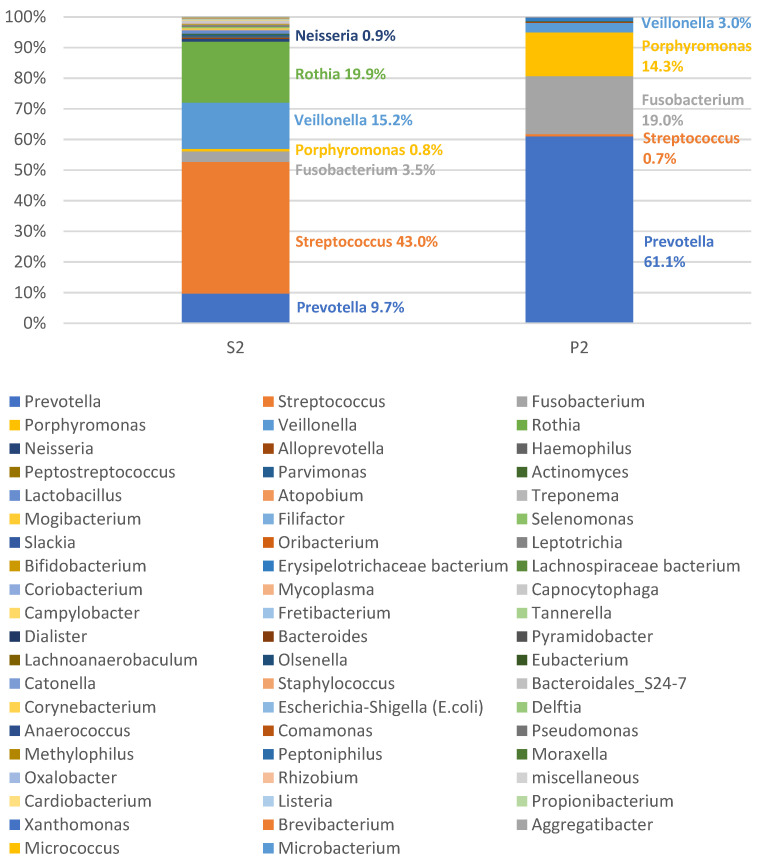
Microbiome of the saliva (S2) and the pus (P2) of patient no. 2. The microbiome of the pus shows the typical picture of a polymicrobial infection. The legend contains all detected bacterial genera of the two related samples. The order in the legend corresponds to the total abundance in saliva and pus of all 50 samples (decreasing from left to right).

Despite the high number of different bacteria even in the pus samples, we found a clear predominance of one genus, namely *Streptococcus,* in two of the 50 (4%) cases. One of these cases is presented in Figure 3. The microbiomes found in the pus of these patients presumably correspond to a bacterial mono-infection with *Streptococcus*, while all other microbiomes of the pus showed the typical picture of a polymicrobial infection without a predominance of a specific genus. Figure 4 outlines a case of a typical polymicrobial infection. Thus, a polymicrobial infection could be observed in 48 of 50 cases (96%).

**Figure 5:**
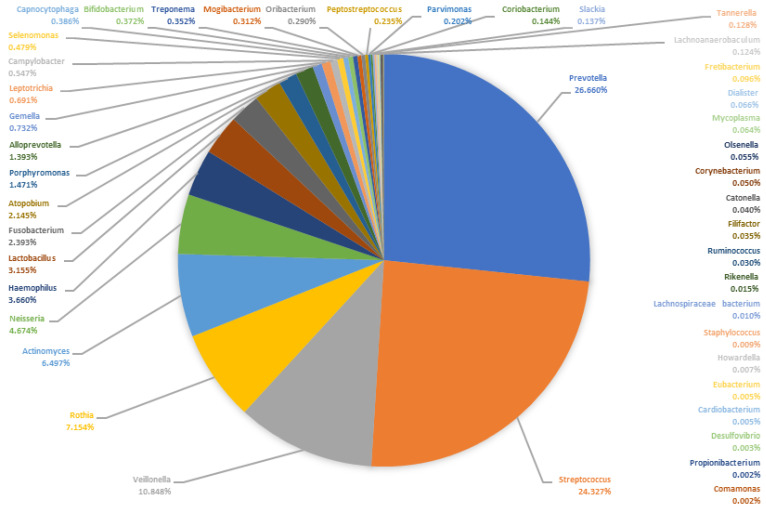
Median microbiome of all saliva samples (*n* = 50). The pie chart shows the proportion of the bacterial genera of the sum of all medians of the relative abundances.

**Figure 6:**
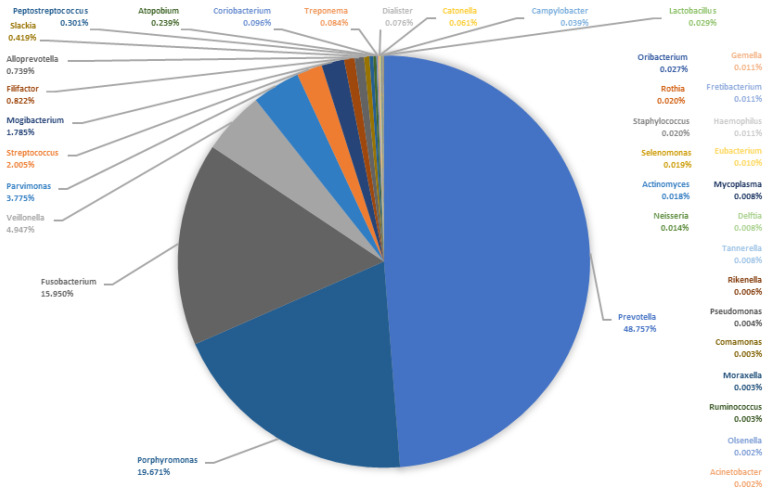
Median microbiome of all pus samples (*n* = 50). The pie chart shows the proportion of the bacterial genera of the sum of all medians of the relative abundances.

In order to show the composition of the microbiomes, Table 1 lists the 50 most frequently detected bacterial genera of the saliva and the pus samples. Since no normal distribution can be assumed for the relative frequencies of the reads, the median can be considered a more suitable measure than the arithmetic mean. Thus, Figure 5 and Figure 6 show the median composition of the saliva and pus microbiomes. It can be clearly seen that the compositions of the microbiomes differ. In particular, the genera *Porphyromonas* and *Fusobacterium* are more common in the pus, while the genus *Streptococcus* is less abundant in the pus. Despite the two described mono-infections with *Streptococcus*, the median relative frequency of the DNA from this genus was only 1.15% in pus.

**Figure 7:**
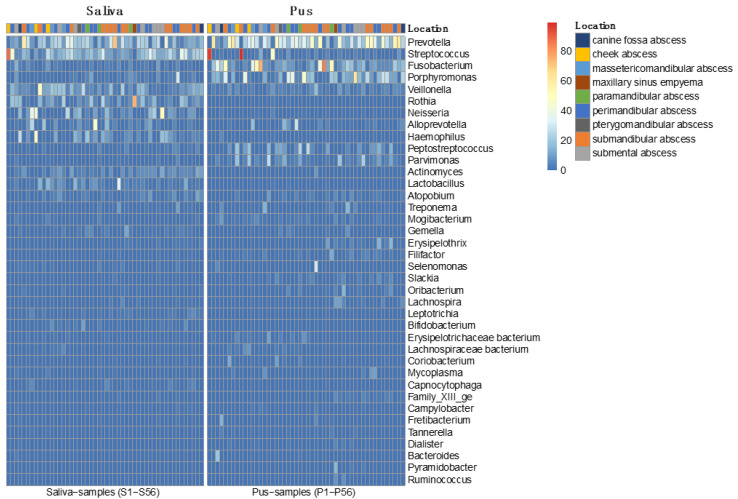
Heatmap of all saliva and pus samples (*n* = 100). Colors show the relative frequencies (sum of rows > 20%). Saliva samples are shown on the left side and pus samples on the right side. It is obvious that the abundance of *Porphyromonas* and *Fusobacterium* is greater in pus while the abundance of *Streptococcus* is greater in saliva.

To describe the composition of the microbiomes in more detail, Figure 7 shows a heatmap of the saliva and pus samples, where the different samples are separated in terms of content (saliva samples on the left side and pus samples on the right side).

**Figure 8:**
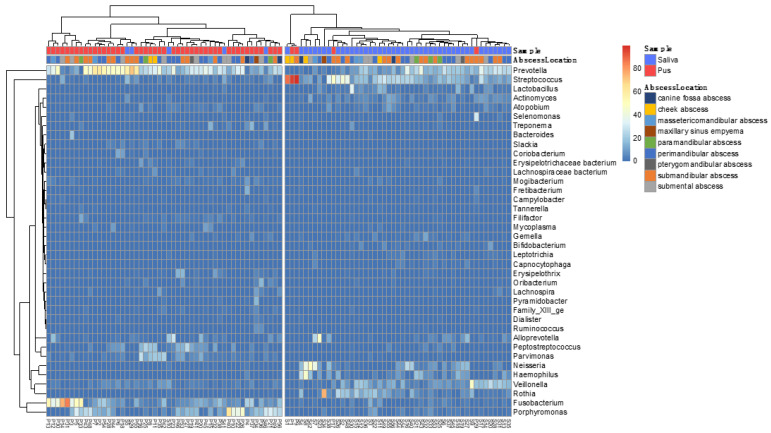
Heatmap of all saliva and pus samples (*n* = 100). Colors show the relative frequency of the reads of the most frequent bacterial genera (sum of rows > 20%). Hierarchical clustering with dendrogram (method: ward with Euclidean distance). It is demonstrated that the samples are almost completely separated by clustering, indicating the different compositions of the samples. The heatmap also shows that abscess location has no significant influence on the composition of the microbiome.

**Figure 9:**
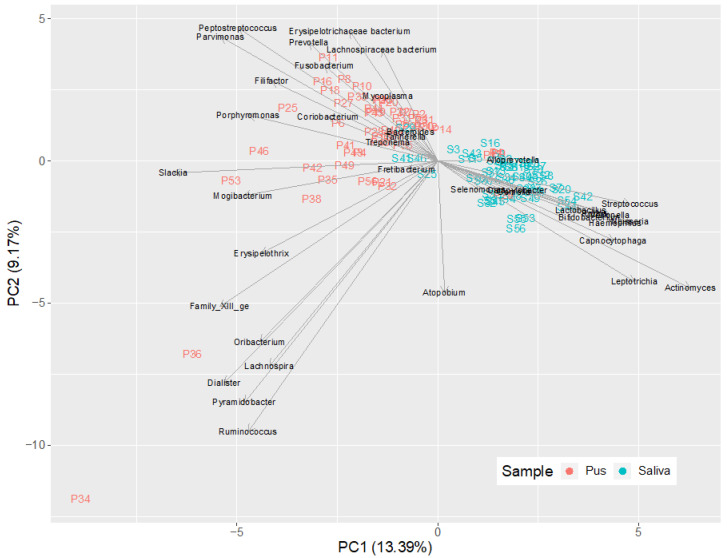
Principal component analysis biplot of all saliva and pus samples (*n* = 100; sum of rows > 20%) in order to visualize the relationship between the bacterial genera and the distances between samples (beta diversity). As can be seen, there is almost a complete separation of the samples (saliva and pus).

Figure 8 shows the corresponding heatmap in which the saliva and pus samples are subjected to hierarchical clustering. It is clearly visible that the algorithm separates the samples almost completely due to the different compositions of the samples. It can also be seen that the abscess location has no influence on the composition of the microbiome. Figure 9 shows this clearly using principal component analysis for the bacteria listed in the heatmap. It is clearly recognizable that the saliva samples are separated from the pus samples, showing the predominant bacteria in saliva at the bottom right and the predominant bacteria in pus at the top left, according to the median microbiomes of saliva and pus (Figure 5 and Figure 6).

**Figure 10:**
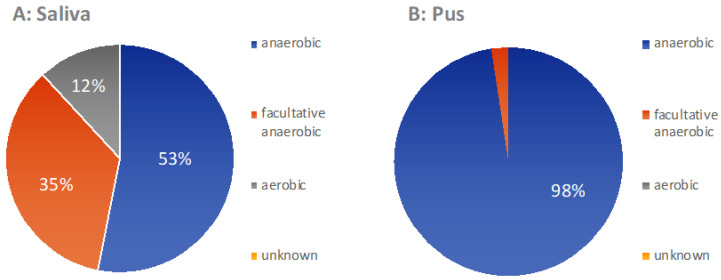
Median metabolism of the saliva samples (**A**) and the pus samples (**B**) (*n* = 50). There is a clear increase in the proportion of anaerobic bacteria in the abscess.

Figure 10 displays the oxygen metabolism of the bacteria in the pus and the saliva according to the median microbiome. It is obvious that the pus group contained predominantly anaerobic bacteria, while, in saliva, around half of the bacteria were aerobic and facultative anaerobic.

**Figure 11:**
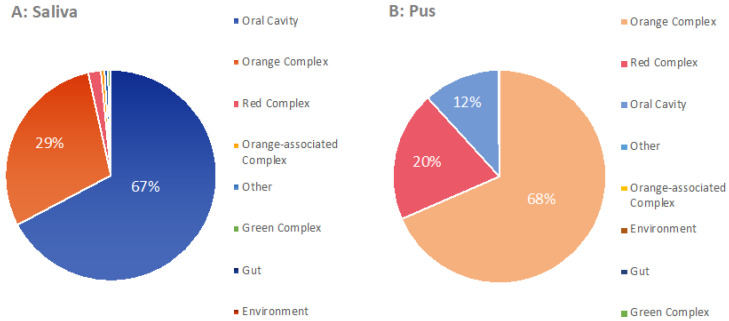
Classification of the median microbiomes ((**A**) saliva (**B**) pus) according to the complexes described by SocranScheme 50. [21]. Bacteria of the red and orange complex are associated with strong pathogenicity in periodontal disease. The genus *Streptococcus* (otherwise yellow complex) was assigned to the “oral cavity” group in this representation.

Regarding the median microbiome of the pus, *Prevotella*, *Porphyromonas* and *Fusobacterium* were especially predominant. Individual species of these genera have been described as highly periodontally pathogenic by numerous authors. Thus, Socransky et al. defined bacterial complexes that are supposed to express different degrees of pathogenicity by means of color [21]. In particular, bacteria that can be assigned to the orange and red complexes indicate strong pathogenicity. With the underlying 16S rRNA gene analysis, the bacteria can only be identified down to the level of the genus, so that a definitive classification cannot be made here. However, Figure 11 attempts to outline a potential affiliation with the orange and the red complex in particular. For the genera with no relationship to the complexes and the genus *Streptococcus* (otherwise “the yellow complex”), the natural habitat of the bacteria is indicated, such as “gut” or “oral cavity”. Considering the results, a possible affiliation to the orange or red complex is observed clearly more frequently in pus than in saliva. It can therefore be assumed that the pathogenic potential of the bacteria identified in pus is significantly higher than in saliva.

**Figure 12:**
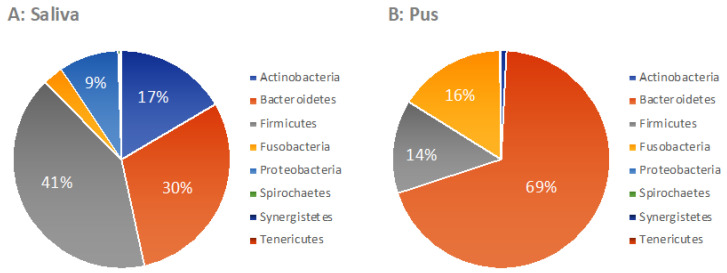
Phylum-level diversity of the oral microbiome (**A**) and the pus microbiome (**B**) (*n* = 50).

Finally, the phylum affiliation of the individual bacterial genera in saliva and pus was determined (Figure 12). In saliva, the results were comparable to those of other investigations [10]. In the pus, the result was also comparable to other studies [3] but with a much clearer predominance of Bacteroidetes.

## 4. Discussion

Acute odontogenous abscesses are among the most common inflammatory diseases in the head and neck region. Most abscesses are localized and can be successfully treated by local incision from the mouth using local anesthesia [3].

Larger abscesses often show a tendency to spread and are usually incised extraorally using general anesthesia [1]. These abscesses can cause severe local and systemic complications and even lead to death [1,2,3]. Local complications are osteomyelitis and sinusitis and systemic complications include sepsis, endocarditis, spondylitis, orbital phlegmon, orbital abscess, necrotizing fasciitis, brain abscess, mediastinitis and adult respiratory distress syndrome [1,3]. Rapid and efficient diagnosis and therapy is therefore of great importance, especially in advanced diseases. In addition to adequate imaging, the determination of the microorganisms present in the abscess is of particular importance.

### 4.1. Culture-Based versus Molecular Detection Methods

Traditionally, culture methods have been used for bacterial detection, which have provided a considerable amount of information on bacterial etiology and the species involved in odontogenic abscesses [3]. Nevertheless, the sole detection of bacteria by means of culture does not seem to provide a fully comprehensive picture of the actually present bacterial spectrum [3]. In order to successfully culture oral bacteria in the laboratory, the culture media must be adapted to their specific variable requirements [12,22]. Since odontogenic abscesses are usually polymicrobial in composition [3], it can be difficult to provide a culture environment that is equally suitable for all relevant bacteria. In addition, bacterial detection may also be incorrect or incomplete due to inadequate transport conditions or delayed start of culture [3]. Early microscopic studies already discussed that the actual number of bacteria in the mouth is probably significantly higher than the number of bacteria that can actually be detected by culture [23]. The introduction of culture-independent molecular biological methods into the analysis of oral bacterial diversity has not only confirmed this picture conveyed by microscopic studies, but also revealed an even broader and more diverse spectrum of oral bacteria [14]. In 2013, Siquera and Rôças described in their review that 40% to 70% of oral bacterial species have not yet been cultivated and phenotypically characterized [3]. To date, a lack of essential nutrients or growth factors in the culture, overfeeding conditions during culture, toxicity of the culture medium itself, inhibition by other microorganisms in the sample and metabolic dependence on other microorganisms have been suggested as possible reasons for the failure of culture-based detection [3]. It is therefore not surprising that only a few species have been described as causative pathogens, especially in culture-based studies on the microbial spectrum of odontogenic abscesses [24,25,26,27,28,29,30]. The review by Siquera and Rôças, for example, reported an average of only 3.3 to 8.5 identified species per abscess using culture-based methods, whereas 77 to 114 genera or species per abscess were found using next-generation sequencing [3]. Despite the widely publicized importance of emergent communities and symbioses [21], the search is ongoing for the “triggering species” to treat effectively with an antibiogram [3]. Numerous authors have demonstrated that endodontic infections such as other endogenous infections are not due to a single bacterial species but usually due to a community of species [31,32,33]. The search for a causative pathogen with the aim of a suitable antibiotic therapy according to an antibiogram therefore appears to be difficult in odontogenic infections.

### 4.2. Microbiome of the Saliva

The aim of this study was to determine the oral microbiome in saliva and the microbiome of the pus of abscesses, respectively, in patients with extensive odontogenic infections using 16S rRNA gene analysis, next-generation sequencing and bioinformatics. For this purpose, a saliva sample was obtained prior to incision of the abscess and a pus sample was harvested during the incision and evaluated beyond normal clinical routine. The examination of the oral microbiome from the saliva samples revealed findings comparable to other examinations [10,34,35,36]. In the review of Verma et al., *Firmicutes* were reported at a maximum of 36.7%, *Bacteroidetes* at 17.1%, *Proteobacteria* at 17.1%, *Actinobacteria* at 11.6%, *Spirochaetes* at 7.9% and *Fusobacteria* at 5.2% [10]. In the present work, the phyla occur with similar frequency (Figure 12), although the frequency of the Spirochaetes was significantly lower. In terms of genus level, several authors have reported an oral core microbiome [10,36]. According to Bik et al. [37], this includes the genera *Actinomyces*, *Atopobium*, *Corynebacterium* and *Rothia* of the phylum *Actinobacteria*; the genera *Bergeyella*, *Capnocytophaga* and *Prevotella* of the phylum *Bacteroidetes*; the genera *Granulicatella*, *Streptococcus* and *Veillonella* of the phylum *Firmicutes*; the genera *Campylobacter*, *Cardiobacterium*, *Haemophilus* and *Neisseria* of the phylum *Proteobacteria* as well as *Fusobacteria* and *TM7*. The genera *Derxia* and *Leptotrichia* were added later by Chen et al. [10,16]. With the exception of the genera *Bergeyella*, *Derxia* and *Granulicatella*, these genera were also detected in the present study in correspondingly relevant frequencies. In contrast, the genera *Chryseobacterium*, *Anaeroglobus*, *Filifactor*, *Lactobacillus*, *Johnsonella*, *Shuttleworthia*, *Brachymonas*, *Propiniovibri, Scardovia*, *Olsenella*, *Cryptobacterium*, *Bulleidia*, *Peptostreptococcus*, *Dialister*, *Gemella*, *Selenomonas*, *Oribacterium*, *Eikenella*, *Kingella*, *Lautropia*, *Propionibacterium* and *Porphyromonas* are classified as variable oral microbiome [10,37]. In the present study, most of these genera also occurred sporadically, but the genera *Porphyromonas* (1.5%) and *Alloprevotella* (1.4%) were also found here with a notable frequency of more than 1% in the median salivary microbiome. The genera *Tannerella* (0.1%), *Parvimonas* (0.2%) and *Filifactor* (0.02%) were also found consistently and in relevant abundance in the saliva of almost all abscess patients. All five genera have been frequently associated with periodontal or apical inflammatory processes [3,38,39] and could be a possible indicator of a more aggressive variable oral microbiome in patients with extensive odontogenic abscesses.

### 4.3. Microbiome of the Pus

As in other previously conducted molecular biological studies on the bacterial spectrum in the pus of odontogenic abscesses, it was shown that the odontogenic infections were usually not only polymicrobial in nature, but that a much higher number of bacteria could be detected by molecular biological methods than by conventional culture-based detection [3]. With a mean number of 31.44 (±12.09) genera in the pus, a separate microbiome of the pus is obvious. There was no case where no bacteria could be detected in the pus. Thus, a situation comparable to a culture-negative abscess could not be observed. In contrast to the polymicrobial nature of odontogenic abscesses, in 2 out of 50 cases, a different scenario emerged, with a strong predominance of only one genus. This was, in both cases, the genus *Streptococcus*, which played a much smaller role in the pus microbiomes of the other 48 samples, as shown in the heatmaps of Figure 7 and Figure 8. It is possible that the molecular detection method in two samples revealed a real mono-infection with a “culprit bacterium”, which probably belonged to normal oral *Streptococcus*. In the remaining 48 samples, however, as in other studies, there was a strong predominance of anaerobic genera, while the facultative anaerobic genus *Streptococcus* played only a subordinate role (Figure 10). The anaerobic genera were, in particular, associated with the development of acute symptoms in apical periodontal lesions in the work of Siqueira and Rôças [3]. The results of the present study thus underline the assumption that mainly bacteria of the genera *Prevotella*, *Porphyromonas*, *Fusobacterium*, *Veillonella* and *Parvimonas* are involved in the formation of acute dentogenic abscesses, even if these bacteria also occur with equal abundance in the root canal in asymptomatic chronic apical periodontitis [40,41]. Principal component analysis (Figure 9) further shows that even bacteria with a lower abundance, such as *Mogibacterium*, *Filifactor*, *Slackia* and *Peptostreptococcus*, may also be characteristic for the microbiological community of an odontogenic abscess.

*Veillonella*, the fourth most common genus of the median pus microbiome, is an early colonizer of oral biofilms and a causative agent of opportunistic infections [42]. In the present study, *Veillonella* shows a similar distribution to the genus *Streptococcus* in both saliva and pus. An interaction with *Streptococcus* in the development of caries [43] as well as the typical presence in the root canal with endodontic infections has been described [44]. Rôças et al. have described in their study that *V. parvula* produces menaquinones that can meet the specific nutrient requirements of *Porphyromonas* and *Prevotella* species for this substance [44]. In addition, *V. parvula* was found to promote the growth of *T. denticola* in co-cultures by providing peptidase activities complementary to those of *T. denticola* [44,45]. The genus *Veillonella* thus appears to be an important member of a pathogenic microbial community together with *Porphyromonas*, *Prevotella* and *Treponema*.

The abundance of the genus *Mogibacterium* is also worth mentioning. With a proportion of 1.8% of the median pus microbiome, the frequency is almost as high as that of the genus *Streptococcus* at 2.0%. Although no direct correlation between odontogenic abscesses and an increased occurrence of *Mogibacterium* has been described so far, there are indeed reports that *Mogibacterium* can occur more frequently in association with pathological processes [46,47]. For instance, the genus *Mogibacterium* is thought to play a role in the development of inflammation in bisphosphonate-associated bone necrosis of the jaw, together with the genera *Porphyromonas* and *Treponema* [48]. Another study reports the frequent occurrence of *Mogibacterium* in the root canal before and after endodontic treatment [49]. *Mogibacterium* may therefore play a greater role in the development of odontogenic abscesses than previously thought. Another genus, the eighth most abundant one, was the genus *Filifactor,* accounting for 0.8% of the median microbiome. For the species *Filifactor alocis*, it has been described that the bacterium, which is very difficult to cultivate, plays a role in both marginal periodontitis and endodontic infection [49,50,51,52]. It is often reported that this pathogen may play a much more important role in the pathogenesis of inflammatory processes than had been assumed in the past [52].

### 4.4. Pathogenicity of the Pus Microbiome

Overall, the results of our study seem to show a clear shift in pathogenicity from the microbiome of saliva to the microbiome of pus. Thus, pathogenicity mechanisms have been reported for many of the genera commonly found in pus [48,53,54,55,56] and the importance of bacterial communities has often been highlighted [3,10,31]. In this context, the bacterial complexes described by Socransky in the development of periodontal diseases in particular became well-known [21,54]. Figure 11 represents an attempt to assign the bacterial genera to a specific habitat or group. Potentially pathogenic bacteria such as *Prevotella*, *Fusobacterum* or *Parvimonas* were therefore assigned to the “orange complex” because of a possible or probable affiliation and genera such as *Porphyromonas*, *Tannerella* and *Treponema* to the “red complex”. The remaining genera were assigned to their natural habitat because of their potentially lower pathogenicity. For example, *Neisseria*, *Haemophilus* or *Streptococcus* were assigned to the “oral cavity” habitat according to their importance in the oral microbiome [10]. Looking at these classifications according to potential pathogenicity, a clear shift towards pathogenicity (orange and red complex) can be seen between their frequency in saliva and their frequency in pus. The microbiome of the abscess is therefore by no means a simple dissipated amount of oral or pharyngeal microbiome. Instead, it is obviously a collection of bacteria and bacterial communities with high pathogenicity.

### 4.5. Phylum-Level Diversity and the Importance of Streptococcus

Looking at the median microbiome of the pus at the phylum level, it can be seen that *Bacteroidetes* (including *Prevotella* and *Porphyromonas*) had by far the largest share of 69%, while *Firmicutes* (including *Streptococcus*, *Veillonella* and *Parvimonas*) had a share of only 14% and *Fusobacteria* had a share of 16%. Compared to other studies based on culture-based methods, the proportion of *Firmicutes* in our work was significantly lower and the proportion of *Bacteroidetes* significantly higher [3,57,58]. In studies based on molecular methods, a lower proportion of *Firmicutes* was also shown, but this was still significantly higher than in our work, at 38% to 64%. [3]. It is remarkable that almost no *Proteobacteria* (*Campylobacter*, *Cardiobacterium*, *Haemophilus*, *Neisseria,* among others) were detected in the abscess by molecular methods, as also seen in this work [3]. It is possible that the detection of these genera by culture is a result of contamination by saliva during sampling. A similar picture emerges looking at the frequently detected genus *Streptococcus*.

In the present molecular study, the genus *Streptococcus* showed a much more dominant occurrence in the salivary microbiome than in the abscess microbiome, which was also confirmed by other authors [10]. However, *Streptococcus* in particular is very frequently detected in pure culture-based detection methods due to their easy cultivation. In contrast, cultivation of anaerobic bacteria is much more delicate, resulting in less frequent detection [24,30,57,59,60,61,62]. It can therefore be assumed that the culture-based detection of *Streptococcus* in odontogenic abscesses, as well as the antibiogram produced by this, may be attributed greater importance than is actually the case. It is quite conceivable that bacteria of the genera *Prevotella*, *Porphyromonas*, *Fusobacterium* and *Parvimonas* are not sufficiently detected by the current culture-based methods and thus no suitable antibiogram can be prepared for the actual “culprit” pathogens of the abscess. This would imply that many culture-based pathogen detection methods for effective therapy adjustment would not only be delayed, but the antibiograms thus generated would even be of questionable relevance. However, predominantly, bacteria of the genera *Fusobacterium* and *Porphyromonas* have been associated with acute exacerbations and strong pathogenicity [3,33,53,55] and frequent resistance to common antibiotics has already been reported for the equally pathogenic bacteria of the genus *Prevotella* [63,64,65]. The question therefore emerges as to whether the solely culture-based evidence, as currently established in many hospitals, is still up to date given the current technical possibilities. In fact, most odontogenic abscesses are probably treated adequately and successfully by early abscess incision and, if there is a tendency to spread, by additional adequate calculated antibiotic therapy even without the availability of an antibiogram [1]. However, particularly in cases of very extensive abscesses that do not respond adequately to therapeutic efforts, adequate bacterial detection with preparation of an appropriate antibiogram can be of critical importance to patient outcomes. For these cases, it is necessary to demand that prompt bacterial determination by molecular biological methods should be introduced as soon as possible as a part of the clinical routine diagnostics.

## 5. Conclusions

1. The oral microbiome of patients with odontogenic abscesses was comparable to that of healthy subjects described in the literature, although very individual. However, the individually variable microbiome could possibly contain more bacteria with increased pathogenic potential.
2. Odontogenic infections are mainly polymicrobial (96%) and rarely mono-infections (4%). Similar to saliva, pus showed its own microbiome, with a mean number of 31.44 (±12.09) genera.
3. Odontogenic abscesses are mainly caused by anaerobic bacterial strains. Aerobic and facultative anaerobic bacteria seem to play a minor role compared to previously published results described by other authors.
4. The most abundant genera in the pus were Prevotella, Porphyromonas and Fusobacterium, followed by Veillonella, Parvimonas, Streptococcus, Mogibacterium and Filifactor.
5. The pus microbiomes likely have a much higher pathogenic potential than the oral microbiomes derived from saliva.
6. Microbiome analysis detects significantly more bacteria than conventional culture-based methods and shows results even in the case of culture-negative samples. Molecular methods are expected to become the gold standard in medical microbiology diagnostics, particularly for polymicrobial infections with a predominance of anaerobic bacteria.

## Figures and Tables

**Table 1:** Relative frequencies in percent of the 50 most frequently detected bacterial genera in the saliva and pus samples, sorted in descending order according to the mean value. It is obvious that the median deviates often from the mean and thus a normal distribution of the data cannot be assumed. The column “S.dev.” represents the standard deviation and the column “MM” extrapolates the median to 100% and indicates what percentage of a saliva or pus sample can be assigned to a corresponding bacterial genus.

Genus (Saliva Samples)	Mean	S.dev	Median	MM	Genus (Pus Samples)	Mean	S.dev.	Median	MM
*Prevotella*	17.65	13.95	16.39	26.67	*Prevotella*	27.12	17.00	27.91	48.76
*Streptococcus*	17.26	13.85	14.95	24.34	*Fusobacterium*	16.03	18.92	9.13	15.95
*Veillonella*	9.33	8.63	6.67	10.85	*Porphyromonas*	12.94	13.95	11.26	19.67
*Rothia*	8.43	11.95	4.40	7.16	*Streptococcus*	6.57	20.07	1.15	2.00
*Neisseria*	7.18	10.99	2.87	4.68	*Peptostreptococcus*	4.91	6.97	0.17	0.30
*Haemophilus*	5.85	8.47	2.25	3.66	*Parvimonas*	4.79	6.58	2.16	3.78
*Actinomyces*	4.39	3.41	3.99	6.50	*Veillonella*	3.31	3.28	2.83	4.95
*Alloprevotella*	4.14	8.01	0.86	1.39	*Alloprevotella*	2.36	5.14	0.42	0.74
*Lactobacillus*	4.10	6.02	1.94	3.16	*Mogibacterium*	1.45	1.78	1.02	1.79
*Porphyromonas*	2.63	4.72	0.90	1.47	*Filifactor*	1.36	2.17	0.47	0.82
*Fusobacterium*	2.35	2.91	1.47	2.39	*Atopobium*	1.32	2.09	0.14	0.24
*Atopobium*	2.23	2.58	1.32	2.15	*Erysipelothrix*	1.28	3.26	0.00	0.00
*Gemella*	1.28	2.05	0.45	0.73	*Slackia*	1.24	1.86	0.24	0.42
*Leptotrichia*	1.14	1.34	0.42	0.69	*Treponema*	1.18	3.10	0.05	0.08
*Bifidobacterium*	1.09	1.97	0.23	0.37	*Oribacterium*	0.99	2.70	0.02	0.03
*Peptostreptococcus*	0.93	1.64	0.14	0.24	*Lachnospira*	0.96	2.34	0.00	0.00
*Capnocytophaga*	0.92	1.46	0.24	0.39	*Erysipelotrichaceae bacterium*	0.85	2.03	0.00	0.00
*Treponema*	0.83	1.96	0.22	0.35	*Selenomonas*	0.82	4.37	0.01	0.02
*Selenomonas*	0.71	1.03	0.29	0.48	*Coriobacterium*	0.79	2.36	0.06	0.10
*Mogibacterium*	0.55	0.96	0.19	0.31	*Family_XIII_ge*	0.74	1.33	0.00	0.00
*Parvimonas*	0.50	0.93	0.12	0.20	*Mycoplasma*	0.71	2.21	0.00	0.01
*Oribacterium*	0.49	0.65	0.18	0.29	*Lachnospiraceae bacterium*	0.66	1.61	0.00	0.00
*Campylobacter*	0.46	0.49	0.34	0.55	*Fretibacterium*	0.51	2.16	0.01	0.01
*Erysipelothrix*	0.42	0.80	0.00	0.00	*Gemella*	0.48	1.42	0.01	0.01
*Lachnospira*	0.41	0.90	0.00	0.00	*Bacteroides*	0.47	2.80	0.00	0.00
*Lachnoanaerobaculum*	0.37	0.66	0.08	0.12	*Pyramidobacter*	0.46	2.24	0.00	0.00
*Lachnospiraceae bacterium*	0.36	0.89	0.01	0.01	*Dialister*	0.41	0.89	0.04	0.08
*Pasteurella*	0.30	0.88	0.00	0.00	*Tannerella*	0.33	0.84	0.00	0.01
*Mycoplasma*	0.29	0.67	0.04	0.06	*Campylobacter*	0.31	0.70	0.02	0.04
*Slackia*	0.25	0.45	0.08	0.14	*Neisseria*	0.31	0.75	0.01	0.01
*Erysipelotrichaceae bacterium*	0.24	0.53	0.00	0.00	*Ruminococcus*	0.30	1.37	0.00	0.00
*Filifactor*	0.24	0.46	0.02	0.04	*Eubacterium*	0.29	0.51	0.01	0.01
*Fretibacterium*	0.24	0.44	0.06	0.10	*Haemophilus*	0.28	0.80	0.01	0.01
*Tannerella*	0.23	0.36	0.08	0.13	*Actinomyces*	0.25	1.21	0.01	0.02
*Family_XIII_ge*	0.23	0.49	0.00	0.00	*Catonella*	0.21	0.58	0.03	0.06
*Coriobacterium*	0.21	0.35	0.09	0.14	*Olsenella*	0.20	0.64	0.00	0.00
*Olsenella*	0.17	0.42	0.03	0.06	*Rikenella*	0.19	0.41	0.00	0.01
*Dialister*	0.14	0.28	0.04	0.07	*Staphylococcus*	0.18	0.79	0.01	0.02
*Ruminococcus*	0.13	0.27	0.02	0.03	*Clostridium*	0.17	0.73	0.00	0.00
*Desulfovibrio*	0.09	0.27	0.00	0.00	*Acidaminococcus*	0.16	0.79	0.00	0.00
*Escherichia-Shigella (E.coli)*	0.09	0.48	0.00	0.00	*Clostridiales bacterium*	0.16	0.52	0.00	0.00
*SR1_(Absconditabacteria)_ge*	0.09	0.37	0.00	0.00	*Bacteroidales_S24-7*	0.16	1.00	0.00	0.00
*Corynebacterium*	0.08	0.15	0.03	0.05	*Peptococcus*	0.13	0.38	0.00	0.00
*Catonella*	0.08	0.14	0.02	0.04	*Rothia*	0.12	0.36	0.01	0.02
*Bacteroides*	0.07	0.38	0.00	0.00	*Stenotrophomonas*	0.12	0.82	0.00	0.00
*Mollicutes*	0.07	0.21	0.00	0.00	*Lactobacillus*	0.12	0.31	0.02	0.03
*Sphaerochaeta*	0.07	0.36	0.00	0.00	*Desulfovibrio*	0.11	0.55	0.00	0.00
*Rikenella*	0.07	0.15	0.01	0.01	*Delftia*	0.09	0.41	0.00	0.01
*Peptococcus*	0.05	0.10	0.00	0.00	*Bacteroidales bacterium*	0.08	0.44	0.00	0.00
*Flavobacterium*	0.04	0.08	0.00	0.00	*Pasteurella*	0.07	0.36	0.00	0.00

## Data Availability

The datasets generated during and/or analyzed during the current study are available from the corresponding author on reasonable request.
